# Cerebral Spinal Fluid levels of Cytokines are elevated in Patients with Metachromatic Leukodystrophy

**DOI:** 10.1038/srep24579

**Published:** 2016-04-15

**Authors:** Kathryn A. Thibert, Gerald V. Raymond, Jakub Tolar, Weston P. Miller, Paul J. Orchard, Troy C. Lund

**Affiliations:** 1 Division of Pediatric Blood and Marrow Transplantation, University of Minnesota, Minneapolis, Minnesota, 55455, US; 2 Division of Pediatric Neurology, University of Minnesota, Minneapolis, Minnesota, 55455, US.

## Abstract

Metachromatic leukodystrophy (MLD) is a lysosomal storage disease resulting from a deficiency of arylsulfatase A causing an accumulation of cerebroside sulfate, a lipid normally abundant in myelin. Sulfatide accumulation is associated with progressive demyelination and a clinical presentation in severe disease forms that is dominated by motor manifestations. Cerebral inflammation may contribute to the pathophysiology of MLD. To date, cytokine levels in the cerebral spinal fluid of MLD patients have not previously been reported. The objective of this study was to evaluate the concentration of inflammatory cytokines in the CSF of patients with MLD and to compare these levels to unaffected controls. Of 22 cytokines evaluated, we documented significant elevations of MCP-1, IL-1Ra, IL-8, MIP-1b and VEGF in the MLD patients compared to unaffected controls. The elevated cytokines identified in this study may play a significant role in the pathophysiology of MLD. Better understanding of the inflammatory and neurodegenerative process of MLD may lead to improved targeted therapies.

Metachromatic leukodystrophy (MLD) is an autosomal recessive lysosomal storage disease caused by a deficiency of arylsulfatase A, a lysosomal enzyme required for the degradation of cerebroside sulfate, a membrane lipid normally abundant in myelin^1^. The resultant lipid accumulation is associated with progressive central and peripheral nervous demyelination. In the severest disease forms, MLD has an estimated birth prevalence of 1.4–1.8 per 100,000^2^,^3^. The disease is classically stratified into 3 forms, depending on the age of symptom onset: late infantile MLD (<3 years), juvenile (3–16 years), and adult (>16). The most common phenotype is late infantile MLD, which is associated with a clinical presentation that is dominated by motor manifestations, including weakness, muscle wasting, muscle rigidity, and gait disturbance^4^. Patients with late-infantile and early juvenile disease typically demonstrate rapid disease progression and death within several years of onset. Hematopoietic cell transplant (HCT) has been shown to slow the disease and appears beneficial in pre-symptomatic juvenile and adult MLD patients, but remains ineffective for the late infantile form^5^,^6^,^7^,^8^. Alternative therapies under exploration include enzyme replacement and gene therapy^9^.

*In vitro* and mouse model experiments have shown inflammatory reactions within the central nervous system, including microglial activation, astrogliosis, recruitment of peripheral macrophages, and the secretion of pro-inflammatory cytokines to be hallmarks of several lysosomal storage diseases^10^. Specifically, *in vitro* sulfatide loading triggers the synthesis and secretion of inflammatory cytokines including TNF-a, IL-1b and IL-8^11^,^12^. These cytokines are thought to amplify tissue damage associated with MLD^12^. To date, however, *in vivo* cytokine levels in patients affected by MLD have not been reported. Here, for this first time, we describe inflammatory cytokine levels in the cerebrospinal fluid and plasma of untreated MLD patients and compare them to healthy controls.

## Results

We analyzed cytokine levels from of CSF and plasma from 8 MLD patients displayed in Table 1. All patients had confirmed MLD with low arylsulfatase A (ARSA) activity and elevated urine sulfatide excretion. All patients had abnormal MRI scans and displayed motor symptoms at the time of evaluation.

We found significantly elevated levels of the following cytokines in the CSF of MLD patients compared to controls as shown in Table 2: MCP-1 at 1006 ± 264 pg/mL vs 330 ± 29 pg/mL (p = 0.0005), IL-1Ra at 51.7 ± 19.7 pg/mL vs. 7.4 ± 5.6 pg/mL (p = 0.009), IL-8 at 79.3 ± 12.3 pg/mL vs. 13.9 ± 1.3 pg/mL (p < 0.0001), MIP-1b at 15.1 ± 5.8 pg/mL vs. 1.7 ± 1.2 pg/mL (p = 0.003), and VEGF at 1.7 ± 0.4 pg/mL vs. undetectable (p = 0.001). No other cytokines differed significantly between MLD patients and controls (data not shown). Plasma values for MLD patients are also shown in Table 2. While our control CSF samples have been validated in the past^13^,^14^, we lacked sufficient control plasma samples in our biorepository. Therefore, the control plasma data in Table 2 shows previously published values from healthy individuals. Berrahmoune *et al*. measured IL-8, MCP-1 and VEGF levels in 304 healthy children (age 4–17 years) and 540 adults (age 18–55 years)^15^. Kleiner *et al*. measured serum IL-1Ra (amongst other factors) in 7 children (age 1–6 years), 30 adolescents (7–17 years) and 35 adults (>18 years)^16^. Using this previously reported data as reference values, the plasma of the MLD patients showed higher levels of cytokines up to 3.3-fold in the case of IL-8 and 7.6-fold in the case of IL-1Ra.

**Figure 1:**
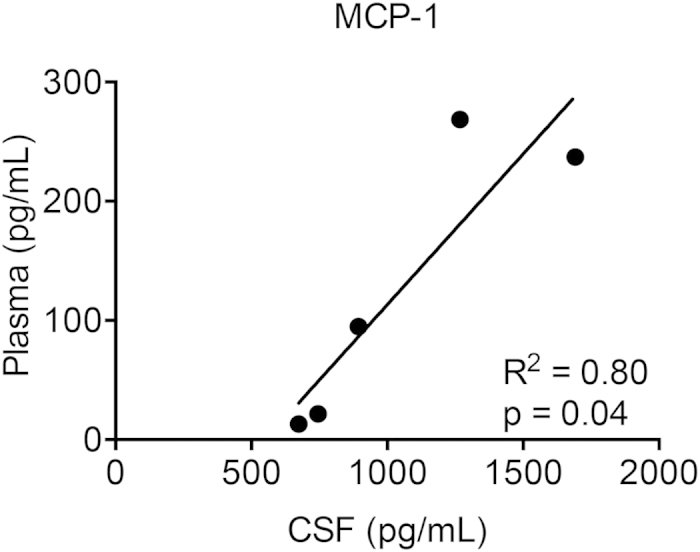
Pearson correlation analysis of CSF and plasma MCP-1 levels in MLD patients. Plasma and CSF samples were drawn at the same time prior to measurement.

**Figure 2:**
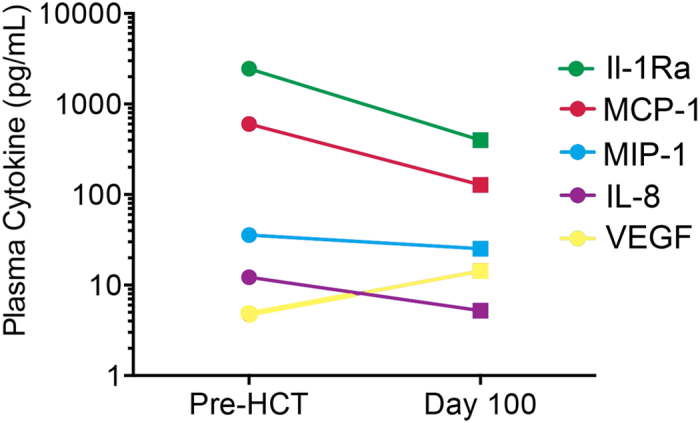
Plasma cytokine profile in one MLD patient prior to HCT and 100 days post HCT.

To learn if cytokines in the CSF correlated to levels in the plasma we analyzed samples that were collected at the same time in five MLD patients (non-synchronous sample were excluded). The only cytokine that showed a significant correlation between CSF and plasma levels was MCP-1 (Fig. 1, R^2^ = 0.80, p = 0.04). Finally, we obtained serial plasma samples from one MLD patient prior to HCT and at 100 days post HCT. Figure 2 indicates that MCP-1, IL-1Ra, IL-8 and MIP-1b were found to be elevated in the plasma prior to HCT, but had decreased by 100 days after HCT. This was not true for VEGF, which was higher 100 days after HCT. While these data are from a single patient, one could speculate that HCT may have led to (partial) attenuation of an inflammatory MLD disease process.

## Discussion

MCP-1, IL-1Ra, IL-8, MIP-1b and VEGF were found to be elevated in the CSF and plasma of patients with MLD compared to controls. Each of the cytokines elevated in our study has different physiological roles and they may be contributing to the pathology of MLD in different ways.

In mouse models of multiple sulfatase deficiency, sulfatide accumulation is associated with increased brain mRNA expression of TNF-a, IL-12 and MIP-1a^17^. In addition, Constantin and Laudanna *et al*. showed exogenous sulfatides incite production of IL-8, IL-1b and TNF-alpha in cultured human monocytes^11^,^12^. Consistent with this finding, we found that IL-8 was significantly higher (approximately 6-fold) in the CSF of patients with MLD. IL-8 is a chemotactic cytokine (i.e. chemokine) capable of activating leukocytes (predominantly neutrophils) after guiding them to sites of inflammation^18^. When activated, neutrophils release phagolysosomal enzymes as well as superoxide and H_2_0_2_, commonly implicated in causing cellular damage^19^.

IL-1Ra is one of three proteins encoded by the IL-1 complex and an IL-1 receptor antagonist. IL-1Ra is typically elevated in inflammatory states and is able to cross the blood-brain-barrier (BBB)^20^. IL-1Ra has been demonstrated to protect neurons from cell death in models of neurodegeneration^21^. Given the elevated levels of IL-1Ra, we can hypothesize the perhaps it is serving as an anti-inflammatory counter-measure in attempt to preserve neurons and glial cells from further damage^22^.

MCP-1 is a central chemokine that recruits monocytes/macrophages to areas of inflammation^23^. A prior murine study used trimethyltin to chemically induce neuronal damage and gliosis (without disruption of the BBB) and determine the pro-inflammatory cytokine signature in response to neuronal damage. The authors found a significant increase in microglial MCP-1 mRNA as early as 2 days post injection, while other pro-inflammatory cytokines were not elevated until 21 days post injection^24^. This suggests that MCP-1 elevation may be an early marker of neuronal damage response in MLD. MCP-1 has also been shown to stimulate the release of lysosomal enzymes and respiratory burst in monocytes. This results in the production of reactive oxygen intermediates that can damage surrounding healthy tissue including myelin^7^. High levels of astrocyte-derived MCP-1 have also been identified in the demyelinated lesions of multiple sclerosis, while being absent in normal white matter^25^. Our finding that CSF and plasma levels of MCP-1 are correlative suggests that a global inflammatory process coexists with a neuroinflammatory one.

Recently, mouse studies have shed light on the importance of inflammation in the pathophysiology of MLD. A study in a non-demyelinating MLD model revealed that extensive microgliosis and increased MIP-1a were present by 24 months of age^26^. This was in contrast to the demyelinating model which showed significant elevations in many inflammatory cytokines (MIP-1a, MIP-1b, IL-9, MCP-1, etc) as early as 10 months of age^26^. It was also shown that the onset of demyelination was preceded by an elevation of MIP-1a followed by upregulation of MIP-1b, MCP-1 and other cytokines^26^. Furthermore, treatment of severe phenotype MLD mice with simvastatin reduced CNS neuroinflammation as evidenced by decreased T-cell infiltration and normalization of levels of MIP-1a. This translated to functional improvement and decreased spinal cord demyelination^26^. We detected elevations of MIP-1b, but not MIP-1a (data not shown) which may represent a fundamental difference in murine and human MLD pathophysiology and should be explored further.

Inflammation also plays a pathophysiologic role in globoid cell leukodystrophy (GLD), a severe lysosomal storage disease resulting from insufficient galactosylceramide β-galactosidase activity. In a mouse model of GLD, astrocytes and microglial cells expressed elevated levels of MCP-1, MIP-1a and MIP-1b that were attenuated following bone marrow transplant^27^. In mice that had the greatest survival following BMT, expression of MCP-1 and MIP-1b was not detectable^27^. These findings support the idea that HCT plays a role in disease attenuation through mediation of inflammation which is consistent with our findings of plasma cytokine attenuation in one MLD patient prior to and following HCT (Fig. 2).

The BBB has remained an obstacle for effective therapy for lysosomal storage diseases in general^28^. VEGF, typically recognized as a potent angiogenic factor, has been shown to disrupt the BBB^29^. It has been shown that VEGF given intravenously to GLD mice at birth can increase the BBB permeability within 2 hours, an effect that persisted at least 24 hours^29^. Furthermore, when VEGF was given before non-ablative HCT without enzyme replacement therapy, the life span of mice with GLD was significantly increased^29^. It is interesting to speculate that the elevations of VEGF we find in MLD CSF and plasma may be contributing to the success of HCT to slow the disease process by allowing neurotropic marrow-derived cells to populate brain parenchyma without the interference of an intact BBB^5^,^8^.

This is the first study to report CSF cytokines in patients with MLD. We determined there were significantly elevated levels of MCP-1, IL-1Ra, IL-8, MIP-1b and VEGF. These cytokines are likely playing a significant role in the pathophysiology of MLD and may result both from sulfatide accumulation or ongoing neuronal damage. Understanding the inflammatory pathways associated with MLD may provide insight for improving disease outcomes.

## Materials and Methods

### Participants

Patients with MLD (n = 1 infantile, n = 4 juvenile, n = 3 adult MLD median age of 15.4 years) underwent lumbar puncture and/or had a blood sample drawn prior to HCT at the University of Minnesota. All patients exhibited abnormally low leukocyte arylsulfatase-A activity and increased urine sulfatide excretion. Control CSF samples were obtained from children (n = 13, median age of 7.4 years) undergoing intrathecal maintenance chemotherapy for a prior diagnosis of acute lymphoblastic leukemia (ALL). All controls were at least 3 months into maintenance therapy and without evidence of central nervous system (CNS) leukemia. The risk of attaining CSF from “healthy children” established the ALL patients as the most appropriate control group available and has been previously published by our group and others^13^,^14^. This study and the use of all biologic samples were approved by the Committee on the Use of Human Subjects in Research at the University of Minnesota. Methods were carried out in accordance with the approved guidelines. Informed written consent was obtained for all patient samples from the parents or guardians on behalf of the child participants. Patient written assent was also obtained if patients were greater than 8 years of age.

### Cytokine Measurements

CSF samples were evaluated using the 22-plex, human panel A (R&D Systems, Minneapolis, MN), measured with the Luminex system (Luminex, Austin, TX), and analyzed by Bioplex software (BioRad, Hercules, CA). This panel includes: epithelial cell-derived neutrophil-activating peptide-78 (ENA-78), basic fibroblast growth factor (bFGF), granulocyte-colony stimulating factor (G-CSF), granulocyte macrophage colony-stimulating factor (GM-CSF), interferon gamma (IFN-gamma), interleukin (IL)-alpha (IL-1a), IL-1beta (IL-1b), IL-1 receptor antagonist (IL-1Ra), IL-2, IL-4, IL-5, IL-6, IL-8, IL-10, IL-17, monocyte chemotactic protein 1 (MCP-1), macrophage inflammatory protein (MIP)-1alpha, MIP-1beta, regulated on activation, normal T expressed and secreted (RANTES) protein, tumor necrosis factor-alpha (TNF-alpha), thrombopoietin (TPO), and vascular endothelial growth factor (VEGF). For each MLD patient and control, samples were analyzed in duplicate and the average value was used for analysis. Plasma samples underwent a targeted analysis for MCP-1, IL-8, VEGF, MIP-1b and IL-1Ra.

### Statistical methods

Means for the MLD and control groups were each calculated and compared with a two-tailed Student’s t-test. Pearson correlation coefficient was calculated between CSF and plasma cytokine levels.

## Additional Information

**How to cite this article**: Thibert, K. A. *et al*. Cerebral Spinal Fluid levels of Cytokines are elevated in Patients with Metachromatic Leukodystrophy. *Sci. Rep*. **6**, 24579; doi: 10.1038/srep24579 (2016).

## Figures and Tables

**Table 1:** MLD patient characteristics.

Patient	MLD Subtype	Age (years)	Sex	ARSA (nmol/hr/mg)	Urine Sulfatides (μg/mg creatinine)	MRI	Symptomatic
1	LI	2.3	F	<1% of normal	4	abnormal	yes
2	LJ	11.1	F	1.9	3	abnormal	yes
3	Adult	42.4	M	“10% of normal”	“elevated”	abnormal	yes
4	Adult	44.2	F	21.8	6.6	abnormal	yes
5	Adult	42.5	F	“low”	6.6	abnormal	yes
6	EJ	5.8	F	8.8	5.9	abnormal	yes
7	LJ	19.6	M	4.1	“excessive”	abnormal	yes
8	EJ	4.5	M	“abnormally low*	“great excess”	abnormal	yes

ARSA and urine sulfatide values or comments were taken from the official diagnostic laboratory report. MLD subtypes: late infantile (LI), late juvenile (LJ), early juvenile (EJ), late juvenile (LJ).

**Table 2:** CSF and plasma cytokine values.

Cytokine	CSF (pg/mL)			Plasma (pg/mL)	
	MLD	Control	P-value	MLD	Reference
MCP-1	987.3 (505.0–1964.0)	330.7 (153.9–632.6)	0.0002	108.1 (13.0–601.8)	77.5 (52.7–113.9)*
IL-8	71.7 (26.3–125)	13.9 (7.3–24.1)	<0.0001	4.4 (1.2–12.2)	1.35 (0.6–2.8)*
VEGF	1.7 (0.0–4.7)	0.0 (0.0–0.0)	0.001	23.8 (4.8–122.5)	28.9 (14.5–57.5)*
MIP-1b	15.1 (0.0–33.7)	1.7 (0.0–15.6)	0.003	n.d.	n.d.
IL-1Ra	51.7 (0.0–136.0)	7.4 (0.0–76.4)	0.009	1282 (161.3–7285.0)	169.2 (134.7–203.6)†

Shown are geometric mean and range for each cytokine.

^*^Represents data from women aged 18–55 years (geometric mean and 1 standard deviation) reported by Berrahmoune *et al*.^15^.

^†^Represents data from Group age 7–17 years (median and interquartile range) reported by Kleiner *et al*.^16^. P-values are derived from a Student’s t-test. Abbreviations: n.d., not done.
